# Bone Regeneration in Load-Bearing Segmental Defects, Guided by Biomorphic, Hierarchically Structured Apatitic Scaffold

**DOI:** 10.3389/fbioe.2021.734486

**Published:** 2021-09-27

**Authors:** Elizaveta Kon, Francesca Salamanna, Giuseppe Filardo, Berardo Di Matteo, Nogah Shabshin, Jonathan Shani, Milena Fini, Francesco Perdisa, Annapaola Parrilli, Simone Sprio, Andrea Ruffini, Maurilio Marcacci, Anna Tampieri

**Affiliations:** ^1^ Department of Biomedical Sciences, Humanitas University, Milan, Italy; ^2^ IRCCS Humanitas Research Hospital, Rozzano, Italy; ^3^ Complex Structure of Surgical Sciences and Technologies - IRCCS Istituto Ortopedico Rizzoli, Bologna, Italy; ^4^ Applied and Translational Research Center, IRCCS Istituto Ortopedico Rizzoli, Bologna, Italy; ^5^ First Moscow State Medical University - Sechenov University, Moscow, Russia; ^6^ Department of Radiology, Emek Medical Center, Clalit Healthcare Services, Afula, Israel; ^7^ Department of Radiology, Pennmedicine, Philadelphia, PA, United States; ^8^ Chavat Daat Veterinary Referral Center, Beit Berl, Israel; ^9^ Hip and Knee Replacement Division, IRCCS Istituto Ortopedico Rizzoli, Bologna, Italy; ^10^ Empa - Swiss Federal Laboratories for Materials Science and Technology, Center for X-ray Analytics, Dübendorf, Switzerland; ^11^ Institute of Science and Technology for Ceramics, National Research Council, Faenza, Italy

**Keywords:** hydroxyapatite, tricalcium phosphate, vascularization, segmental bone defect repair, biomimetism, ion doping, biomorphic scaffold

## Abstract

The regeneration of load-bearing segmental bone defects remains a significant clinical problem in orthopedics, mainly due to the lack of scaffolds with composition and 3D porous structure effective in guiding and sustaining new bone formation and vascularization in large bone defects. In the present study, biomorphic calcium phosphate bone scaffolds (GreenBone™) featuring osteon-mimicking, hierarchically organized, 3D porous structure and lamellar nano-architecture were implanted in a critical cortical defect in sheep and compared with allograft. Two different types of scaffolds were tested: one made of ion-doped hydroxyapatite/β-tricalcium-phosphate (GB-1) and other made of undoped hydroxyapatite only (GB-2). X-ray diffraction patterns of GB-1 and GB-2 confirmed that both scaffolds were made of hydroxyapatite, with a minor amount of β-TCP in GB-1. The chemical composition analysis, obtained by ICP-OES spectrometer, highlighted the carbonation extent and the presence of small amounts of Mg and Sr as doping ions in GB-1. SEM micrographs showed the channel-like wide open porosity of the biomorphic scaffolds and the typical architecture of internal channel walls, characterized by a cell structure mimicking the natural parenchyma of the rattan wood used as a template for the scaffold fabrication. Both GB-1 and GB-2 scaffolds show very similar porosity extent and 3D organization, as also revealed by mercury intrusion porosimetry. Comparing the two scaffolds, GB-1 showed slightly higher fracture strength, as well as improved stability at the stress plateau. In comparison to allograft, at the follow-up time of 6 months, both GB-1 and GB-2 scaffolds showed higher new bone formation and quality of regenerated bone (trabecular thickness, number, and separation). In addition, higher osteoid surface (OS/BS), osteoid thickness (OS.Th), osteoblast surface (Ob.S/BS), vessels/microvessels numbers, as well as substantial osteoclast-mediated implant resorption were observed. The highest values in OS.Th and Ob. S/BS parameters were found in GB-1 scaffold. Finally, Bone Mineralization Index of new bone within scaffolds, as determined by micro-indentation, showed a significantly higher microhardness for GB-1 scaffold in comparison to GB-2. These findings suggested that the biomorphic calcium phosphate scaffolds were able to promote regeneration of load-bearing segmental bone defects in a clinically relevant scenario, which still represents one of the greatest challenges in orthopedics nowadays.

## Introduction

The regeneration of load-bearing segmental bone defects is today considered among the greatest challenges in orthopedics, due to the lack of biomaterials effective for long bone substitution and to the frequent occurrence of various complications such as nonunions (Giannoudis and Atkins, 2007).

In the last 20 years, there have been numerous attempts in the development of new materials for the treatment of long bone defects, particularly based on hydroxyapatite (HA) and other calcium phosphates (CaPs), which most closely resemble the mineral composition of natural bone (Habraken et al., 2016). However, despite preclinical promising results, the clinical application of these materials in various forms and with different physico-chemical features is still restricted for long bone defects consequent to excision of tumors, infection, major trauma, and nonunion (Ebrahimi et al., 2017; Roffi et al., 2017). Indeed, currently available CaP-based devices for large bone defects often fail in recreating the anatomical and functional features of the lost tissue, due to the complexity of bone in terms of composition, structural, and mechanical properties (Hutmacher et al., 2004). This is a critical issue when it comes to regenerate load-bearing segmental bones where multiaxial biomechanical stresses are particularly demanding, so that insufficient structural organization of the newly formed bone tissue can result in impaired functionality. In this respect, the typical osteon structure of the long bone has a multi-scale hierarchical architecture which is highly functional in ensuring: 1) the propagation of mechanical forces from the macro- to micro-scale and 2) the activation of mechano-transduction processes responsible for the bone self-repair ability.

Hence, the main obstacle to the regeneration of critical segmental bone defects is the lack of technologies enabling the fabrication of 3D porous scaffolds capable to recreate *in vivo* the complex ensemble of cell-instructive physico-chemical, topological, and structural signals inducing the formation and remodeling of mechanically competent bone tissue in large, load-bearing defects. To meet this goal, such a scaffold should have several different properties, difficult to achieve at the same time: 1) bioactive chemical composition, 2) bio-resorbability, 3) interconnected, cell-conducive porous architecture to enable ossification and vascularization in the whole scaffold volume, and 4) bone-mimicking mechanical performance promoting mechano-transduction phenomena and bone remodeling from woven to mature, mechanically competent tissue (Huang and Ogawa, 2002; LeGeros, 2002; Hannink and Arts, 2011; Stegen et al., 2015). Particularly, when dealing with inorganic materials such as CaPs, the need of a sintering process for scaffold consolidation yields to phase degradation and crystal growth which impair bioactivity and bio-resorbability. In addition, sintered CaPs are usually characterized by brittle fracture behavior, which is a concern for their use in load-bearing models due to the risk of sudden rupture (Ginebra et al., 2018). Previous studies attempted to solve this problem by developing ceramic-polymer composites, but in spite of the advantage of limiting the occurrence of fragile fracturing, the regenerative ability of such devices is limited by the lack of compositional and mechanical affinity with natural bone, and by the delivery of harmful by-products through nonenzymatic acidic dissolution, which easily induce inflammatory reactions, thus damaging bone cells at the implant site and reducing the bone tissue regeneration process, particularly in the presence of large bone defects (Liu et al., 2006).

In the attempt to overcome the limitations inherent to the classical approach for bio-ceramics fabrication, recent studies reported innovative nanotechnological processes based on the biomorphic transformation of natural wood structures into 3D porous CaP ceramics showing hierarchical architecture (Tampieri et al., 2009; Tampieri et al., 2018; Filardo et al., 2020). In particular, rattan wood was used as a template for such a process based on its bone-like hierarchical structure mimicking the osteon structure typical of the long bone (Tampieri et al., 2009; Tampieri et al., 2018; Filardo et al., 2020). Previous studies in 3D bioreactor showed that, in comparison with sintered hydroxyapatite scaffolds featuring similar porosity extent, biomorphic scaffolds made of hydroxyapatite/tricalcium phosphate doped with Mg and Sr ions are capable of triggering dramatic overexpression of genes involved in osteogenesis and to promote cross-talk between osteoblasts and fibroblasts, thus potentially stimulating angiogenesis (Tampieri et al., 2018; Sprio et al., 2019). Furthermore, the scaffolds were found to have mechanical performance unusual for a pure ceramic material, resulting in high anisotropic compressive and tensile strength, high toughness and bone-like, damage-tolerant behavior, making them very promising for application as bone substitutes in load-bearing bone defects (Tomlinson et al., 2001; Bigoni et al., 2019).

In the present work, the performance of a scaffold (GreenBone™), produced via biomorphic transformation of rattan wood structures, was tested in a sheep model of segmental bone loss and compared to that of an allograft implant. The purpose of the work was to assess the regenerative ability of the scaffold in a load-bearing site and to show whether the enhanced biological efficiency and mechanical ability previously demonstrated *in vitro* can be confirmed in a clinically relevant animal model.

## Materials and Methods

### Scaffolds Preparation

The scaffolds tested in this work were obtained by a biomorphic transformation process as previously reported (Tampieri et al., 2018), using cylindrical rattan wood pieces (*Calamus manna*) as 3D templates guiding the process. Briefly, the wood template was pyrolyzed at 1,000°C under nitrogen gas flow to obtain a biomorphic (i.e., reproducing the original wood structure) carbon template, then placed in a vessel containing metallic Ca granules (Sigma Aldrich, United States), and subjected to heating up to 1,250°C to allow sublimation of calcium in an oxygen-free environment, and reaction with the carbon template to obtain a 3D biomorphic calcium carbide (CaC_2_). The resulting CaC_2_ was converted into calcium oxide (CaO) by heating at 900°C under air atmosphere. Conversion of the resulting CaO into a calcium carbonate (CaCO_3_) template was carried out by thermal treatment under a flux of carbon dioxide under non-isothermal and isobaric conditions (*p* = 100 atm) under dry or wet conditions from room temperature to 800°C. To activate CO_3_ ↔ PO_4_ ion exchange in the as-obtained CaCO_3_ template, it was placed in a closed reactor containing a 2.0M solution of (NH_4_)_2_HPO_4_ (Sigma Aldrich, United States ) and heated at 220°C under water vapor pressure (∼20 bar) to obtain a biomorphic CaP scaffold. Then, a conclusive treatment was carried out by soaking the obtained scaffold in an aqueous solution containing 0.5M Sr(NO_3_)_2_ and 0.5M Mg(NO_3_)_2_ ions for 24 h at 50°C, thus obtaining a Mg, Sr-doped biomorphic scaffold (hereinafter coded as GB-1). The undoped biomorphic scaffold is hereinafter coded as GB-2.

### Scaffolds Characterization

The phase composition of the scaffolds was obtained by X-ray diffraction (XRD), using a D8 Advance diffractometer (Bruker Karlsruhe, Germany) equipped with a Lynx-eye position sensitive device (CuKα radiation: λ = 1.54178 Å). XRD spectra were recorded in the 2θ range of 20–60° with a counting time of 0.5 s and a step size of 0.02°. XRD spectra were subjected to full profile analysis (TOPAS software v.5, Bruker, Karlsruhe, Germany) to evaluate cell parameters and coherent domain sizes.

Chemical analysis was performed on dried samples using an ICP-OES spectrometer (Agilent 5,100). About 20 mg of the sampling material was dissolved in 2 ml of nitric acid (Sigma Aldrich; 65 vol%) and then diluted with Milli-Q water to obtain 100 ml of solution. The solution was then analyzed using a standard prepared from primary standards (1,000 ppm, Fluka). The carbonate content was evaluated by thermogravimetric analysis (Stanton STA 1500, Stanton, London, United Kingdom), measuring the weight loss in the temperature range 600–1,100°C.

Mercury porosimetry was used to evaluate pore size distribution (<50 μm) by two different equipment (Carlo-Erba Porosimeter 2000 and Macropores Unit 120), working on separate pore size ranges. The open and total porosities of the studied ceramics were measured by Archimede’s method and geometrical weight volume evaluation, respectively.

The compressive strength of the scaffolds was measured on cylindrical specimens sized 20 × 14 mm (height × diameter) using a Zwick/Roell instrument, model Z050 (Ulm, Germany). Microscopic evaluation of the scaffold was made by field emission-scanning electron microscopy (FEG-SEM) (Sigma NTS GmbH, Carl Zeiss, Oberkochen, Germany) in order to evaluate the porous architecture of the scaffolds at the multi-scale.

#### Scaffold Liquid Absorption

The scaffold’s liquid absorption ability was evaluated by soaking into body fluid. Five dried scaffolds (three GB1 and two GB2) were weighted (0.1 mg accuracy) (SI-64, Denver Instrument GmbH) and then immersed in 10 ml of sheep blood for 1 min at room temperature. After this procedure, the scaffolds were placed for a 10 s on a qualitative filter paper disk to remove the blood in excess, and the weight was measured again. The liquid absorption was calculated with the following equation:

$$LiqA=\frac{m_{2}-m_{1}}{m_{1}}\times 100$$

where *LiqA* is the blood absorption (%), *m*_*1*_ is the dry mass, *m*_*2*_ is the wet mass.

#### Mechanical Stability of the Scaffold

The scaffold’s mechanical stability was examined by the drill test. Ten scaffolds were drilled by using the orthopedic power tool Colibri II (DePuy Syntheses GmbH; Oberdorf—Switzerland), using a drill bit of 2.5-mm diameter, to place a 3.5-mm diameter cortex self-tapping screw. During this procedure, digital photos of each scaffold were taken, and the images were elaborated with Adobe Photoshop software v.6.0.1.

### *In vivo* Study in Sheep Model

The study was approved by the institutional Committee for Ethical Conduct and Care and Use of Laboratory Animals of Israeli Ministry of Health (approval 09/2016). Study procedures were carried in accordance with the requirements set out in the Animal Welfare Requirements Part 2, Animal Welfare Law, the Guide for Care and Use of Laboratory Animals by the Institute of laboratory Animal Resource (ILAR), the guidelines by the National Institute of Health (NIH), and by the Association For Assessment And Accreditation Of Laboratory Animal Care (AAALAC), and in accordance with Parts 1, 2, and 6 of regulations for Biological evaluation of medical devices. The study has herein been reported following the ARRIVE guidelines.

#### Study Design

The present study was performed as a joint research between the Assaf-Harofeh Medical Center (Israel) and the IRCCS Istituto Ortopedico Rizzoli (Bologna, Italy) experimental facilities. The study evaluated the safety and performance of both GB-1 and GB-2 against allograft transplantation in the repair of a critical bone defect in 24 healthy female Assaf-Awassi sheep, 59–90 kg in weight. After selection, animals were housed in a dedicated enclosure at least 5 days prior to the surgical operation, where they could roam freely. Feeding was withheld the day before surgery.

The sheep were randomized into three study groups to receive either the scaffold GB-1, GB-2 or the allograft (AG, control group). Implants for the AG group were obtained from the bone segments removed from both GB-1 and GB-2 animal groups. Bone segments removed from the animals were washed with saline and antibiotics and conserved in a refrigerator for less than 48 h.

After implantation, the animals were closely monitored: follow-up procedures consisted of daily inspection of the animals, a weekly health assessment by a certified veterinarian, a monthly radiographic evaluation, and CT scans at 3 and 6 months. Animals were sacrificed at the end of the 6 months follow-up period.

#### Surgical Procedure

Surgeries were performed in aseptic conditions. Sheep were placed on the right side, and the right hind limb was shaved and disinfected. The metatarsus shaft was exposed through a medial approach directly above the bone to reach the medial side. A 3.5 mm broad dynamic compression plate with eight holes was contoured to the shaft, and holes were drilled with a high-speed perforator: three distally and three proximally to the defect. A standardized 2 cm defect was created with an oscillating saw between the fourth and fifth screw hole, under constant irrigation, while preservation of soft tissues was obtained by using two retractors. The 2-cm bone segment with periosteum was then removed, and after implant insertion, the plate was fixed to the bone with 3.5 mm screws, and the soft tissues were closed in layers. The correct positioning of the implant and plate was ensured using intraoperative fluoroscopy.

The lesion gap was treated according to the following groups:

Group 1— biomorphic scaffold GB-1
Group 2— biomorphic scaffold GB-2
Group 3— allograft (AG).

Removed bone segments from the animal’s Group 1 and 2 were conserved in a refrigerator (−20°C) for 24–48 h and used as allograft implants in Group 3.

After wound dressing, the animals were anesthetized and anterior–posterior and medial-lateral X-rays were performed by means of an X-ray generator (6.4 mA; 55 kV). Then, a full cast was put on the operated leg. The sole of the claws was closed for 4 weeks and then left open to ensure weight-bearing while preventing torsional or shear forces on the fracture site through the cast immobilization of the metatarsus shaft. The cast was changed at 4 weeks or upon need, and at 8 weeks, the sheep were let out to graze.

#### Anesthesia and Postoperative Medication

Prior to the surgery, the animals were subjected to sedation *via* an intramuscular injection of ketamine (19 mg/kg) and xylazine 2% (1 mg/kg). Subsequent anesthesia was induced by intravenous injection of midazolam (0.1–0.5 mg/kg or to effect). The animals then underwent endotracheal intubation using an AG-EET. Anesthesia was maintained using isoflurane (1–3% with 100% O_2_) and aided by mechanical ventilation. Postoperative medication included antibiotics for at least 2 weeks following implantation (cefazolin 1 gr bid). Analgesic administration included dipyrone (1 gr bid on operation day) and flunixin (2.2 mg/kg/day, 1M) for the first 2 weeks following surgery.

#### Euthanasia

Euthanasia was carried out under general anesthesia and performed via intravenous injection of potassium chloride, in accordance with facility procedures. After euthanasia, a segment from the operated leg (which included the scaffold and an additional 3 cm of bone above and below it) was removed for subsequent analyses.

#### Radiography and CT Scan

Implant performance was assessed monthly by means of blind radiographic assessment in the antero-posterior (AP) and lateral planes (6.4 mA; 55 kV) at months 1, 2, 4, 5, 6 plus by CT scans including coronal and sagittal reformats (Siemens, Polymobil III, Philips ICT256) at months 3 and 6 by an independent radiologist with 17 years of experience. The implant performance and the healing process were quantified following the modified RUST scoring system, which focuses on bridging callus formation and fracture appearance, obtained as a cumulative score of single scores. In detail, the following parameters were evaluated: 1) callus formation, 2) new bone formation, and 3) implant resorption. The scoring system for callus formation ranged from 1 to 3: 0, no callus formation; 1, non-bridging; 2, partial bridging callus; and 3, complete bridging callus. New bone formation referred to the medullary cavity and was evaluated on CT as a high-density material in the medullary canal. It was scored as 0, none; 1, some; and 2, massive new bone formation. Finally, implant resorption was evaluated on both radiographs and CT, with the scores indicating 0, none; 1, partial; 2, complete resorption. The highest possible score was given if the boneimplant was barely seen.

#### Macroscopic Assessments

On the same day in which the animals were euthanized, regional lymph nodes of each animal were harvested and macroscopically examined to detect any alteration of the normal structure. Furthermore, popliteal lymph nodes were fixed in 4% formaldehyde within 1 h following euthanasia, dehydrated in a graded series of alcohols, and finally, embedded in paraffin. Histopathological evaluation examined: subcapsular sinus, follicles, germinal centers, high endothelial venules of cortex and medullary cords, and the sinus of the paracortex medulla.

Tissue reaction was assessed in terms of nature and extent of any of the following: hematoma, edema, encapsulation, and/or additional gross findings; presence, shape, and location of implant including possible remnants of degradable material; lymph node presentation.

#### Micro-CT

Architectural characteristics and measurements were assessed in a non-destructive manner by micro-CT at postmortem dissection and plate removal. Explanted metatarsus bone segments containing implanted scaffolds or AG were scanned with a high-resolution microtomography system (Skyscan 1,176, Bruker Micro CT, Belgium) at 80 kV and 300 μA using a copper and aluminum filter. The nominal resolution (pixel size) was set for all samples at 17.50 μm. The acquired images were then reconstructed using the NRecon software (Bruker MicroCT, Belgium) and were corrected for alignment (depending on the acquisition), beam hardening, and ring artifact reduction. The resulting images were saved in jpg 8-bit format. Quantitative 3D analyses were carried out with CTAn software (Bruker Micro CT, Belgium, v.1.16.4.1) on all samples. Volumes of interest (VOIs) of 20 mm or 40 mm in height and 35 mm × 35 mm on the axial plane were defined in each sample, using the implanted material as reference. Morphological parameters evaluated were:

- nBV (in mm^3^), newly formed bone volume;
- Cl.V (in mm^3^), callus volume at the defect level;
- Ct. V in %, cortical volume,
- Ct. Th in mm, cortical thickness;
- Tb.Th (in mm), trabecular thickness of the newly formed bone;
- Tb.N (in mm^−1^), trabecular number of the newly formed bone;
- Tb. Sp (in mm), trabecular separation of the newly formed bone.

#### Histology and Histomorphometry

To evaluate the local histopathological response, metatarsi were dehydrated in ethyl alcohol solutions of increasing concentration (one passage at 70%, two passages at 95%, and two passages at 100%) in intervals of 24–48 h per solution. The samples were then soaked in methyl methacrylate. After polymerization, the blocks containing the samples were sectioned along a plane parallel to the long axis of the implant (EXAKT Cutting Systems, GmbH and Co., Norderstedt, Germany). From each sample, 5–0 and 100–200 µm sections were obtained. Sections were evaluated by an optical microscope (Olympus BX51), and two of them were selected for further histological evaluations. The two selected sections were attacked on a special microscope slide (Microscope Slides, 50 × 100 × 1.5 mm, EXAKT); the precise adherence of the sections to the acrylic glass was performed with a cyanoacrylate glue which allows a complete adhesion in about 20–30 min. After 1 day, the sections were thinned with a grinding system (550, ATM GmbH, Mammelzen, Germany) using abrasive papers of different granulation (Struers), from 600 to 4,000 grit, up to a thickness of 40 ± 10 µm. Before staining, sections were treated with a polycrystalline diamond spray and polished automatically with a polishing system (Saphir 550 Grinding/Polishing System). The two sections were stained with toluidine blue/fast green and mounted with a cover glass using a mounting medium (contains acrylic resin and xylenes).

Histological and quantitative histo-morphometric analyses were carried out with digital scanner with a resolution of 0.5 μm (CS System, Aperio Technologies, Vista, CA—United States) and with an optic microscope (Olympus BX51). Regions of interest (ROIs) of 20 and 5 mm in height were defined in each sample, by using the implanted material as reference. Parameters assessed included changes in tissue morphology, quality of bone ingrowth, material fragmentation, presence of debris, shape and position of the implanted materials, extent of fibrous capsule (Fb.Ar), and inflammation–as compatible with methyl methacrylate embeddingpresence/extent of necrosis, presence of calcified tissue (osteoid surface, OS/BS), thickness of non-calcified tissue (osteoid thickness, Os.Th), bone surface covered by osteoblasts (Ob.S/BS).

To further assess inflammation (polymorphonuclear cells, lymphocyte plasma cells, macrophages) and neovascularization (number of vessels/microvessels) of the implanted materials, a region of interest (ROI) of 1 cm × 1 cm, on the lateral side of the metatarsus (opposed to the osteosynthesis plate), was obtained from two unstained sections. The obtained sections were attacked on a microscope slide (as previously described) and thinned down to a thickness of 15 ± 3 µm. These sections were stained with Stevenel’s blue/picro-fuchsin and mounted with a cover glass using a mounting medium. Histological and quantitative histo-morphometric analyses were carried out as previously described with a digital scanner and with an optic microscope. The histological and histo-morphometric parameters assessed were inflammation (i.e., presence of polymorphonuclear cells, lymphocytes plasma cells, macrophages, giant cells) and neovascularization (vessels/microvessels numbers).

#### Microhardness

Bone maturation, mineralization, and mechanical strength of newly formed bone were evaluated with the Vickers indentation test in the polymerized blocks containing the scaffolds (sectioned along a plane parallel to the long axis of the implant, as described above), and in 6 non-implanted scaffolds embedded in methyl methacrylate. This test allows evaluation of resistance of bone to indentation which can reflect biochemical proprieties such as mineral content. The microhardness evaluations were performed through a Vickers indentation test using a microdurometer (DuraScan 70; EMCO-TEST Prüfmaschinen, Kuchl, Austria) connected to an optical microscope, which also allows to identify and differentiate the newly formed bone, formed by thin and dense trabeculae with new osteonic systems, from the preexisting host bone, cortical bone formed by dense and compact bone. Microhardness measurements were performed perpendicularly to the surface with a Vickers indenter (quadrangular pyramid with an angle of apical 136° 15′) applying a load of 0.05 Kg and for a time of 5 s.

The index of bone microhardness (HV) was calculated by dividing the indentation strength for the imprint surface on the bone and observed under the microscope (Table 1). In the non-implanted scaffolds, the microhardness average value was calculated on a mean of 10 measurements. The microhardness average value for each metatarsus was calculated on a mean of 10 measurements for each examined area: 1) pre-existing host bone, 2) bone at the scaffold and AG interface, 3) scaffold/AG, and 4) newly formed bone. Finally, the bone mineralization index (BMI) was calculated as a percentage by dividing the HV of newly formed bone by the HV of preexisting host bone, as reported in Table 1.

**TABLE 1:** Microhardness parameters.

Parameter	Description
The index of bone microhardness (HV)	Calculated by dividing the indentation strength for the imprint surface on the bone and observed under the microscope
Bone mineralization index (BMI %)	$\mathrm{BMI}=\frac{\mathrm{HV of new bone}}{\mathrm{HV of host bone}}\times \mathrm{100}$

HV, Vickers Hardness Index; BMI, Bone Mineralization Index.

### Statistical Analyses

Statistical analysis was performed using R v.3.3.3 software (R Team, 2002). The significant level considered was α = 0.05. For dependent variables, the normal distribution (ShapiroWilk test) and homogeneity of variance (Levene test) of data were evaluated. Data are reported as box-plot graphs or histograms representing mean ± SD, at a significant level of *p* < 0.05.

Data from imaging (X-ray and CT) were analyzed with one-way ANOVA. Data on vessels and microvessels number were analyzed with nonparametric KruskalWallis test followed by MannWhitney U test. The other *ex-vivo* and microhardness data of scaffolds were analyzed with a one-way ANOVA by considering “scaffold” factor—three levels (GB-1, GB-2, and AG)—as fixed effect. When *ex-vivo* data violated the assumption of homogeneity of variances, Welch’s ANOVA was used. Two-way ANOVA was used to analyze bone microhardness results by considering factors “scaffold” and “measurement site”—three levels (host bone; newly formed bone within scaffold; and cortical bone at the interface with scaffold)—as fixed effects. Then, the post hoc pair-wise comparison Tukey HSD test was used to compare data among groups. Finally, the Bonferroni correction of *p*-value was applied rejecting the null hypothesis for each *p* ≤ *α*/*m*, where α is the desired overall alpha level and m the number of hypotheses, in order to control the family-wise error rate.

## Results

### Scaffolds Preparation and Characterization

**FIGURE 1:**
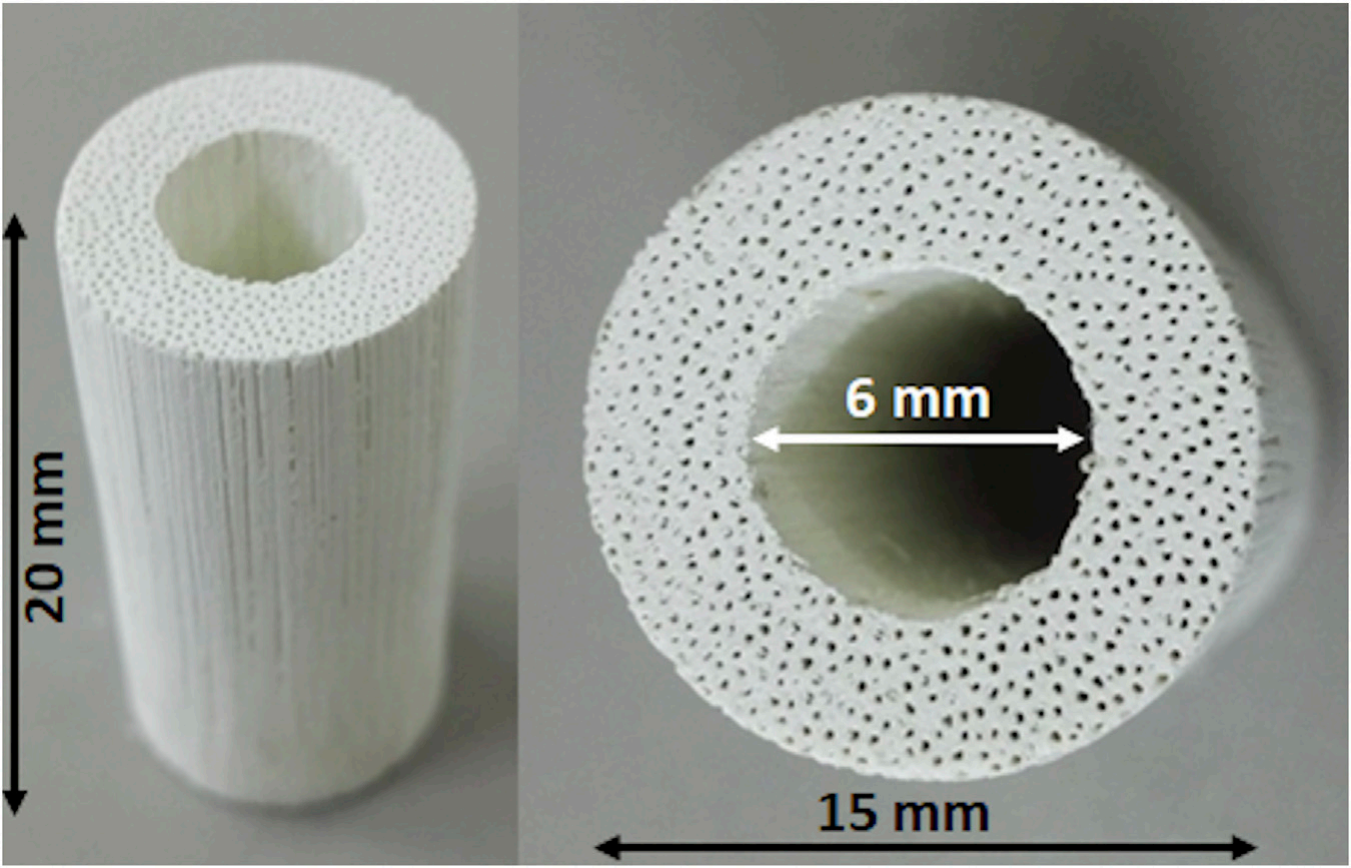
Macroscopic images of the GB scaffold.

**FIGURE 2:**
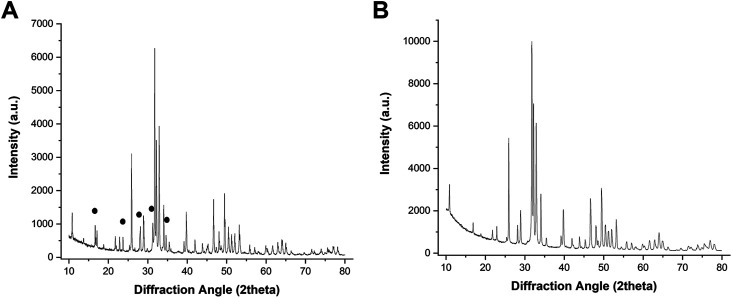
XRD spectra of GB1 **(A)** and GB-2 **(B)**. The black signs point to major XRD reflections belonging to the TCP phase.

Figure 1 shows a macroscopic view of the GB scaffold, highlighting the channel-like porosity running throughout the whole scaffold length, as well as the central hole, created to mimic the medullary cavity of long bones and to improve new bone penetration and vascularization *in vivo*. Figure 2 shows the X-ray diffraction patterns of GB-1 (Figure 2A) and GB-2 (Figure 2B), showing that both scaffolds are made of hydroxyapatite, with a minor amount of β-TCP in GB-1. In this respect, Table 2 shows the semiquantitative phase composition of the scaffolds, as well as the chemical composition obtained by ICP-OES spectrometer, highlighting the carbonation extent and the presence of small amounts of Mg and Sr as doping ions in GB-1.

**TABLE 2:** Composition and porosity extent of GB-1 and GB-2 scaffolds.

Scaffold type	GB-1	GB-2
	HA + β-TCP	HA only
	—	—
Phase composition	—	—
HA	85 ± 10 vol%	100%
β-TCP	15 ± 10 vol%	0%
	—	—
Chemical composition	—	—
Ca/P molar ratio	1.6 ± 0.1	1.6 ± 0.1
Mg	< 1.5 wt%	—
Sr	< 1.5 wt%	—
CO_3_	< 8 wt%	—
	—	—
Physical properties	—	—
Shape	cylinder	cylinder
Total porosity	>45%	>45%
Micropore size <10 μm	>30%	>30%
Macropore size >10 μm	>30%	>30%

**FIGURE 3:**
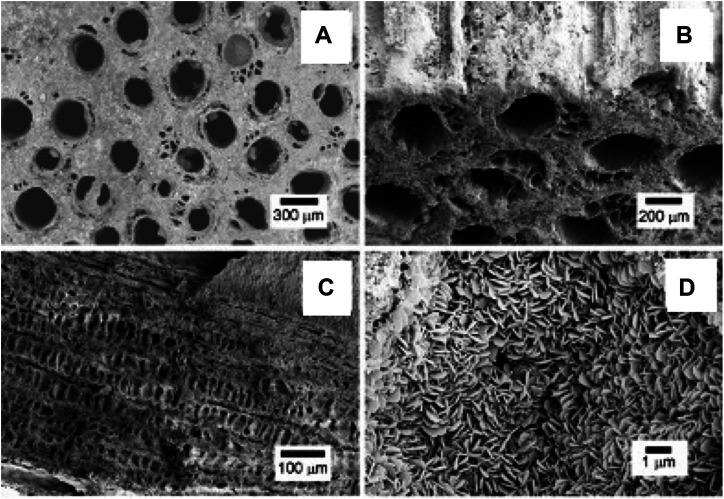
SEM micrographs showing: **(A)** the upper scaffold surface showing large channel-like pores, better evidenced in **(B)** where a different tilt angle shows the external channel walls; **(C)** a longitudinal section of the scaffold showing the internal channel wall, revealing a cell-like pore structure interconnecting the different large channels; **(D)** a detail of the scaffold structure, showing its constituting building blocks, made of nano-sized hydroxyapatite lamellae.

SEM micrographs in Figure 3 show the typical hierarchical structure and pore morphology of biomorphic scaffolds at different size scales. In particular, Figures 3A,B show the channel-like wide open porosity of the biomorphic scaffolds, running throughout the whole scaffold, whereas Figure 3C shows the typical architecture of internal channel walls, characterized by a cell structure mimicking the natural parenchyma of the rattan wood used as a template for the scaffold fabrication [17]. Figure 3D shows a detail of the nano-size lamellar structure characterizing both the biomorphic scaffolds. Both GB-1 and GB-2 scaffolds show very similar porosity extent and 3D organization, as also obtained by mercury intrusion porosimetry (Table 2).

**FIGURE 4:**
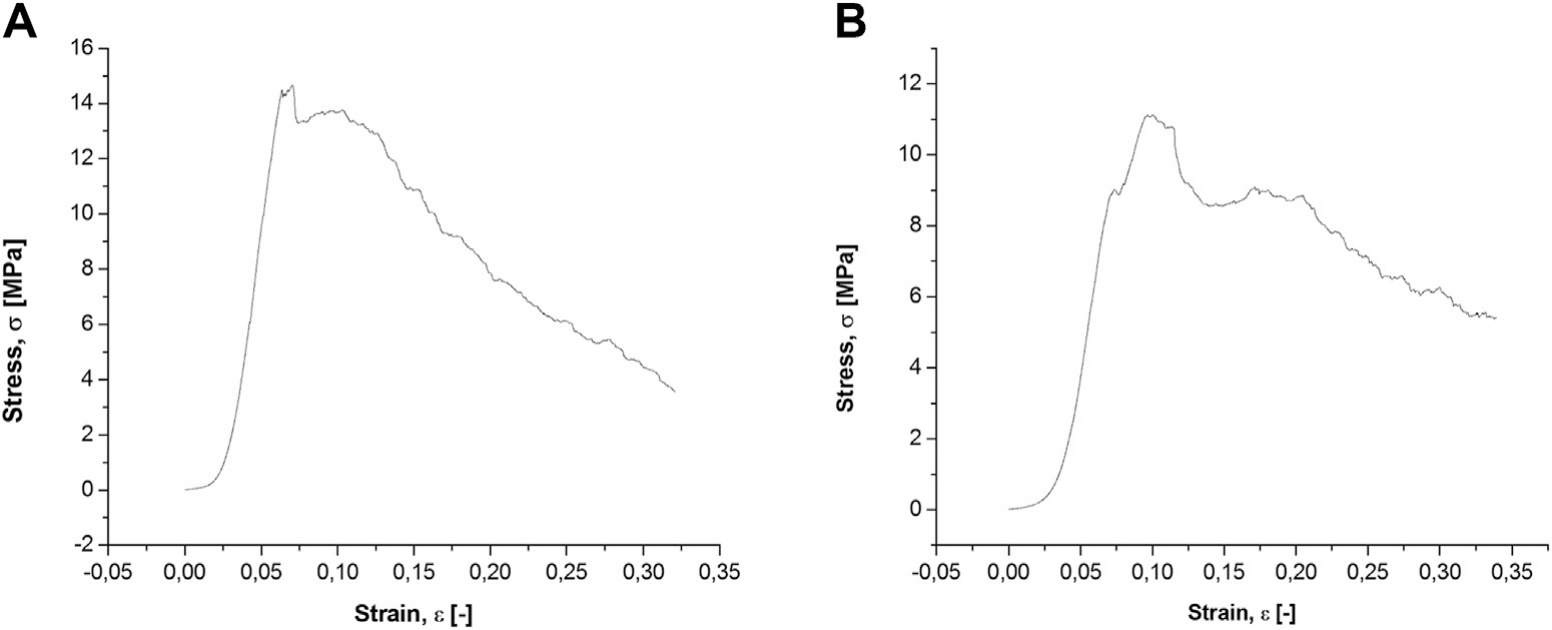
Compressive strength analysis on GB-1 **(A)** and GB-2 **(B)** scaffolds.

The mechanical behavior of the biomorphic scaffolds, tested under compression along the direction of the main channels (Figure 4), shows, after a linear elastic stage, a stress plateau in the range 11–15 MPa where the scaffold starts to fracture but maintaining good structural integrity up to a deformation of ∼30%. Such a damage-tolerant behavior is quite unusual for pure ceramic materials and can be ascribed to the hierarchical porous architecture of the biomorphic scaffolds that facilitates dissipation of the mechanical forces, thus preventing sudden rupture, similarly as occurs in bone (Gibson et al., 1999). Comparing the two scaffolds, GB-1 shows slightly higher fracture strength: particularly we measured compressive strength values of 14.8 ± 1.2 MPa and 11.1 ± 1.4 MPa for GB-1 and GB-2 scaffold, respectively, as well as improved stability at the stress plateau for GB-1 than for GB-2.

#### Scaffold Liquid Absorption

The scaffold’s ability to absorb fluids permits to evaluate the capacity to support the nutrient and metabolite transfer between the material and the surrounding tissue. Results of the liquid absorption analyses revealed the ability of the scaffolds to absorb blood; no significant differences were seen between GB-1 and GB-2 (LiqA (%): GB1 = 12.06 and GB2 = 12.82; *p* = 0.4748).

### *In vivo* Safety and Performance

#### Macroscopic Assessments

All animals survived surgery and follow-up. No treatment-related changes in the lymph nodes (specifically, the popliteal lymph nodes) were observed. One sheep developed infection on the screws, followed by progressive loosening of the proximal screws and plate deformation, so it was excluded from the evaluation. No serious adverse events occurred. Of the initial 24 animals, 23 were evaluated at the final follow-up.

#### Radiography and CT Scan

**FIGURE 5:**
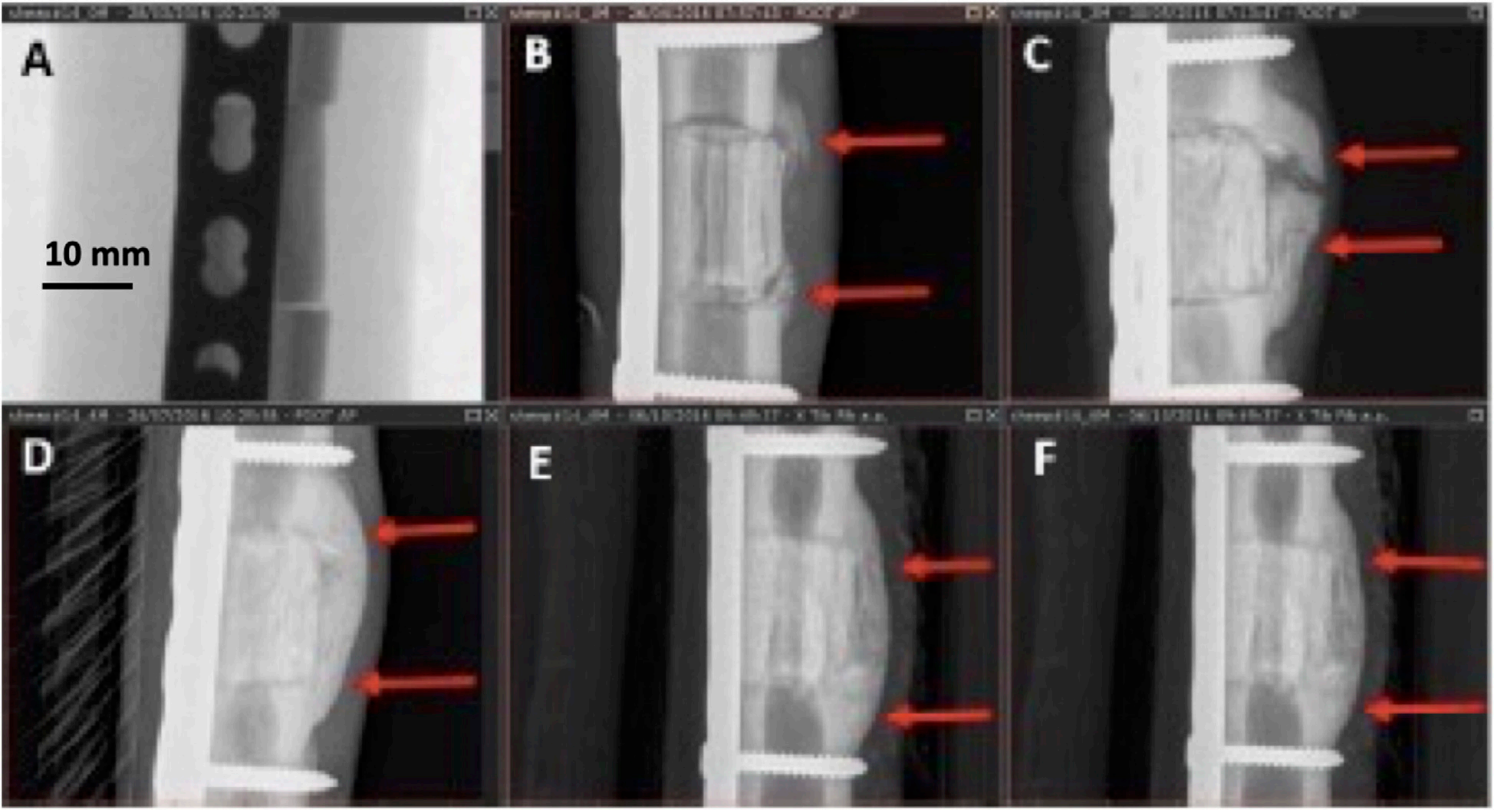
Different stages of callus formation (GB-1). Serial radiographs on the same sheep. **(A)**: immediate post implant radiograph (score 0), **(B)**: month 1, non-bridging callus (score = 1), **(C)**: month 2, partially bridging callus (score = 2), **(D)**: month 4, complete bridging callus (score = 3), **(E,F)**: months 5, 6, complete bridging unchanged (score = 3).

**FIGURE 6:**
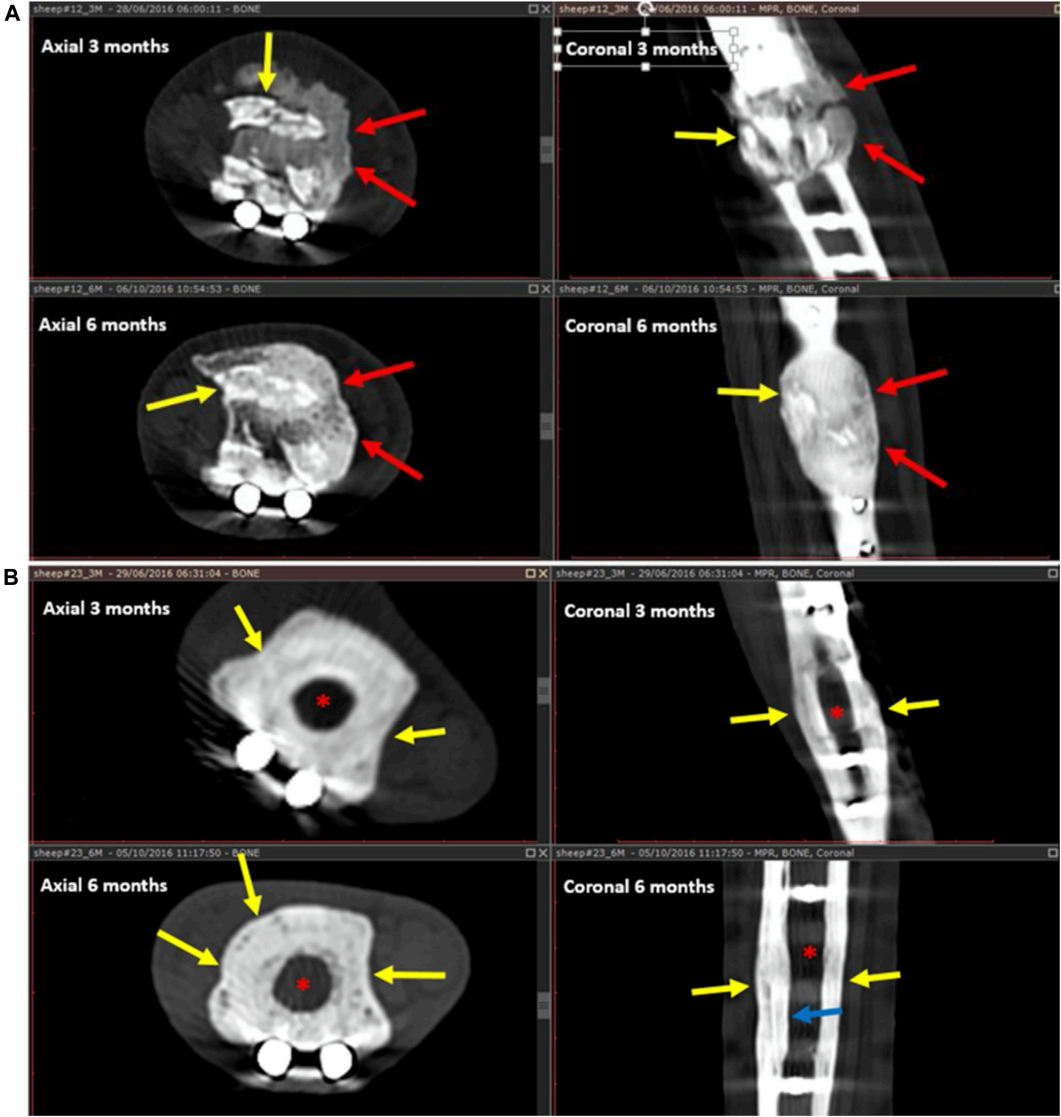
New bone formation and resorption for GB-1 scaffold **(A)** (and AG) **(B)**. A: Healing after GB-1 scaffold. At 3 months **(upper** images), there is already remarkable bone formation seen as a calcified matrix filling the medullary canal (red arrows). There is also implant resorption seen as fragmentation of the implant (yellow arrows). At the 6 months follow-up CT **(lower** images), the resorbing implant fragments (yellow arrows) are well incorporated into the newly formed bone (red arrows). The new bone appears more mature, and there is a suggestion of a trabecular pattern. B: Healing after AG placement on months 3 and 6 CT follow-up. After 3 months **(upper** images), there is callus formation encircling the allograft (yellow arrow). The medullary canal remains unchanged, without new bone formation (red asterisk). After 6 months, the AG becomes osteopenic, barely seen, is surrounded by the callus (yellow arrows), and there is mild endosteal thickening (blue arrow). The medullary canal (red asterisk) is not involved in the healing process and remains unchanged.

Callus formation was equally observed across all samples with no significant difference among the three groups (Figure 5). In addition to cortical healing and callus, new bone formation (seen as calcified matrix at CT) was observed at the medullary cavity for both GB-1 and GB-2 scaffolds but not in the AG group in which such pattern was not observed (medullary canal remained unchanged) (GB-1 and GB-2 *vs* AG *p* < 0.001). In particular, the GB-1 group featured new bone formation at an earlier time-point, which was already visible at month 3; by month 6, new bone formation appeared comparable between GB-1 and GB-2 scaffolds (Figure 6).

Implant resorption was only observed for GB-1 and GB-2 scaffolds and not for the AG and was clearly visible at month 1 from implant (Figure 6). Resorption at CT and radiography appeared as gradual fragmentation of the implant, which evolved alongside new bone formation and with no significant differences among GB-1 and GB-2 (Figure 6). Osteotomy lines (at proximal and distal portions of implant) evaluated at month 6 were not detectable in samples of GB-1, slightly detectable (incomplete healing) in GB-2, and visible in AG (Figure 6).

#### Micro-CT

Concerning bone parameters measured at each selected VOI, significant differences were found between GB-1 and GB-2 in comparison to AG, whereas no significant differences were detected between GB-1 and GB-2 scaffolds (Table 3).

**TABLE 3:** Results (ANOVA and Tukey HSD test) of microtomographic parameters.

	VOI = h20 mm	Inside material	VOI = h20 mm callus defect	VOI = h40 mm
nBV (mm³)	—	—	—	—
One-way ANOVA	F=44.97 *p* < 0.0005	F=32.97 *p* < 0.0005	F=12.62 *p* < 0.0005	F=31.11 *p* < 0.0005
Tukey HSD test	—	—	—	—
GB-1 vs GB-2	*Ns*	*Ns*	*Ns*	*Ns*
GB-1 vs AG	*p* < 0.0005	*p* < 0.0005	*p* < 0.0005	*p* < 0.0005
GB-2 vs AG	*p* < 0.0005	*p* < 0.0005	*p* = 0.004	*p* = 0.004
Tb.Th (mm)	—	—	—	—
One-way ANOVA	F=0.23	F=58.64 *p* < 0.0005	F=2.64 *ns*	F=0.29 *ns*
	*Ns*	—	—	—
Tukey HSD test	—	—	—	—
GB-1 vs GB-2	*Ns*	*ns*	*Ns*	*ns*
GB-1 vs AG	*Ns*	*p* < 0.0005	*Ns*	*ns*
GB-2 vs AG	*Ns*	*p* < 0.0005	*ns*	*Ns*
Tb.N (1/mm)	—	—	—	—
One-way ANOVA	F=14.34 *p* < 0.0005	F=1.67 *ns*	F=13.09 *p* = 0.0014	F=16.91 *p* < 0.0005
Tukey HSD test	—	—	—	—
GB-1 vs GB-2	*Ns*	*ns*	—	Ns
GB-1 vs AG	*p* < 0.0005	*ns*	*p* = 0.0007	*p* < 0.0005
GB-2 vs AG	*p* = 0.002	*ns*	*p* = 0.036	*p* = 0.0046
Tb.Sp (mm)	—	—	—	—
One-way ANOVA	F=41.59 *p* < 0.0005>	F=41.42 *p* < 0.0005>	F=20.27 *p* < 0.0005	F=32.10 *p* < 0.0005
Tukey HSD test	—	—	—	—
GB-1 vs GB-2	*Ns*	*ns*	*ns*	*Ns*
GB-1 vs AG	*p* < 0.0005	*p* < 0.0005	*p* < 0.0005	*p* < 0.0005
GB-2 vs AG	*p* < 0.0005	*p* < 0.0005	*p* < 0.0005	*p* < 0.0005

Cortical volume (Ct.V, %) and thickness (Ct.Th, mm) measured in VOI of 40 mm did not show any significant differences among groups (Ct.V: F = 1.22, *p* = 0.316—GB-1: 4.95 ± 0.95, GB-2: 5.58 ± 0.75, AG: 5.82 ± 1.45; Ct. Th: F = 0.16, *p* = 0.85—GB-1: 1.20 ± 0.33, GB-2: 1.29 ± 0.23, AG: 1.27 ± 0.46).

#### Histology and Histomorphometry

**FIGURE 7:**
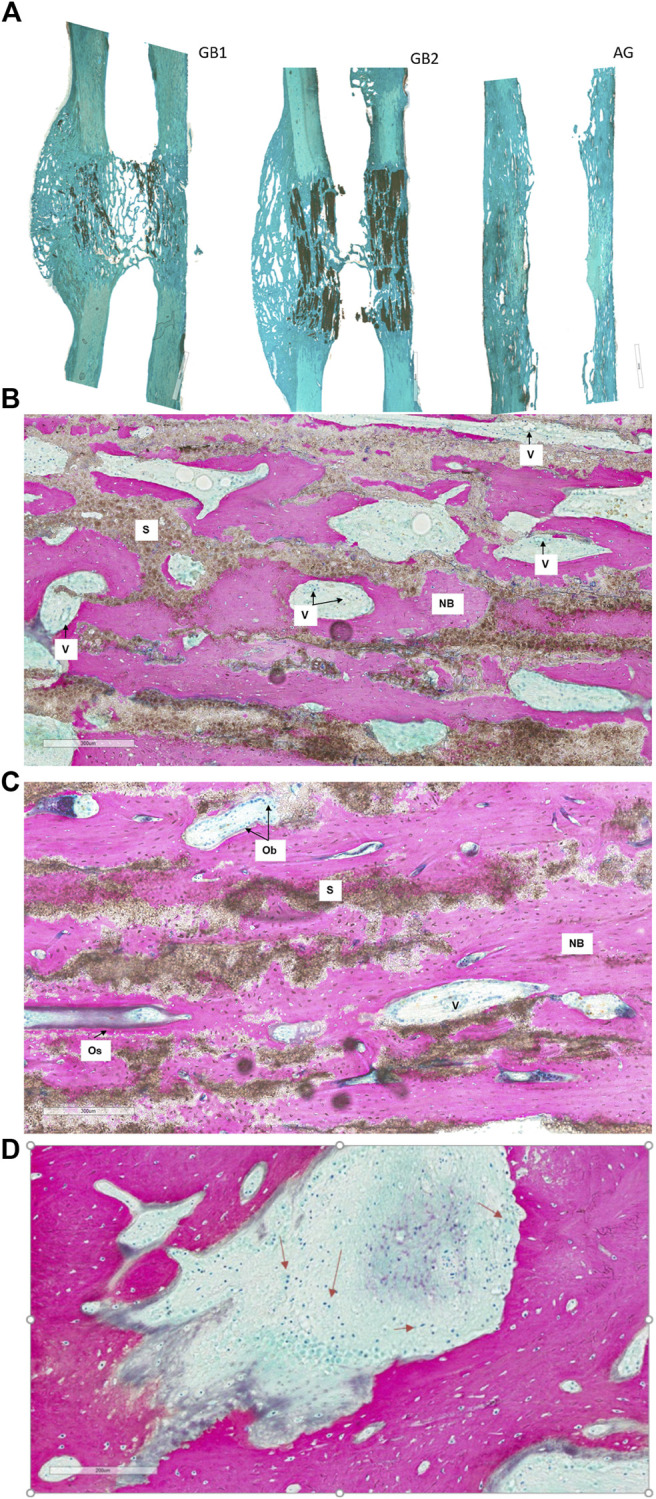
Histological images of GB-1, GB-2, and AG groups 6 months after reconstruction of a critical cortical defect in sheep. **(A)** A representative overview of GB-1 and GB-2, AG groups. Toluidine Blu/Fast Green stain. Magnification ×0.4. **(B)** GB-1 Group, **(C)** GB-2 Group. **(D)** presence of lymphocytes and macrophages among the trabeculae of new bone (arrows). NB: new bone; S: scaffold; V: vessels; Ob: osteoblast; Os: osteoid. Stevenel’s blue/picro-fuchsin stain. Magnification ×8.

Histological evaluation showed neither fibrous encapsulation nor inflammatory processes at the bone-scaffold interface for all groups. Some inflammatory cells (i.e., foreign body giant cells, macrophages, and lymphocytes) were observed, as expected following the aforementioned surgical procedure (Figure 7).

**FIGURE 8:**
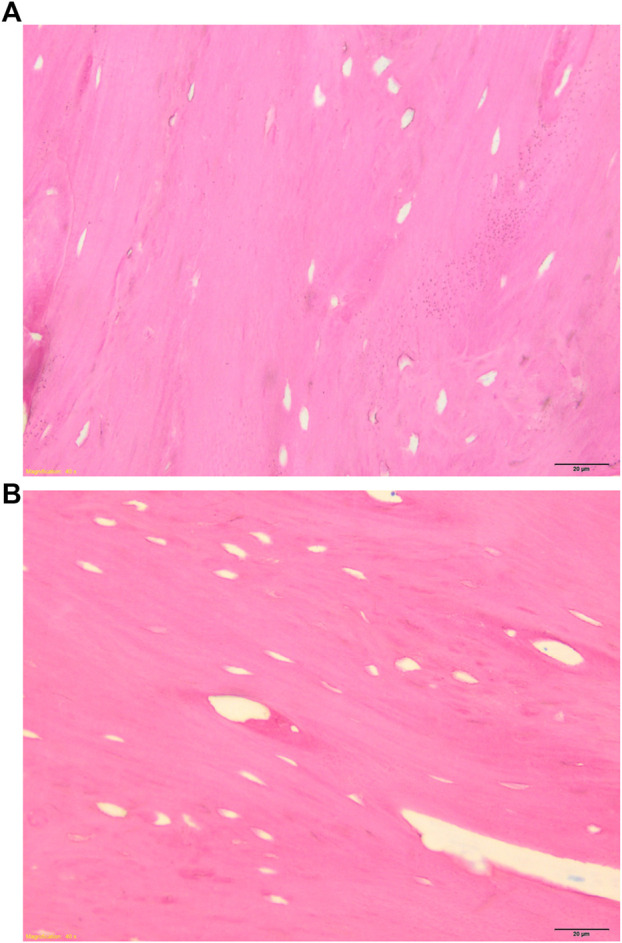
Histological images of AG group 6 months after reconstruction of a critical cortical defect in sheep. **(A)** Magnification ×8, **(B)** Magnification ×40. Stevenel’s blue/picro-fuchsin stain. Magnification ×8.

**FIGURE 9:**
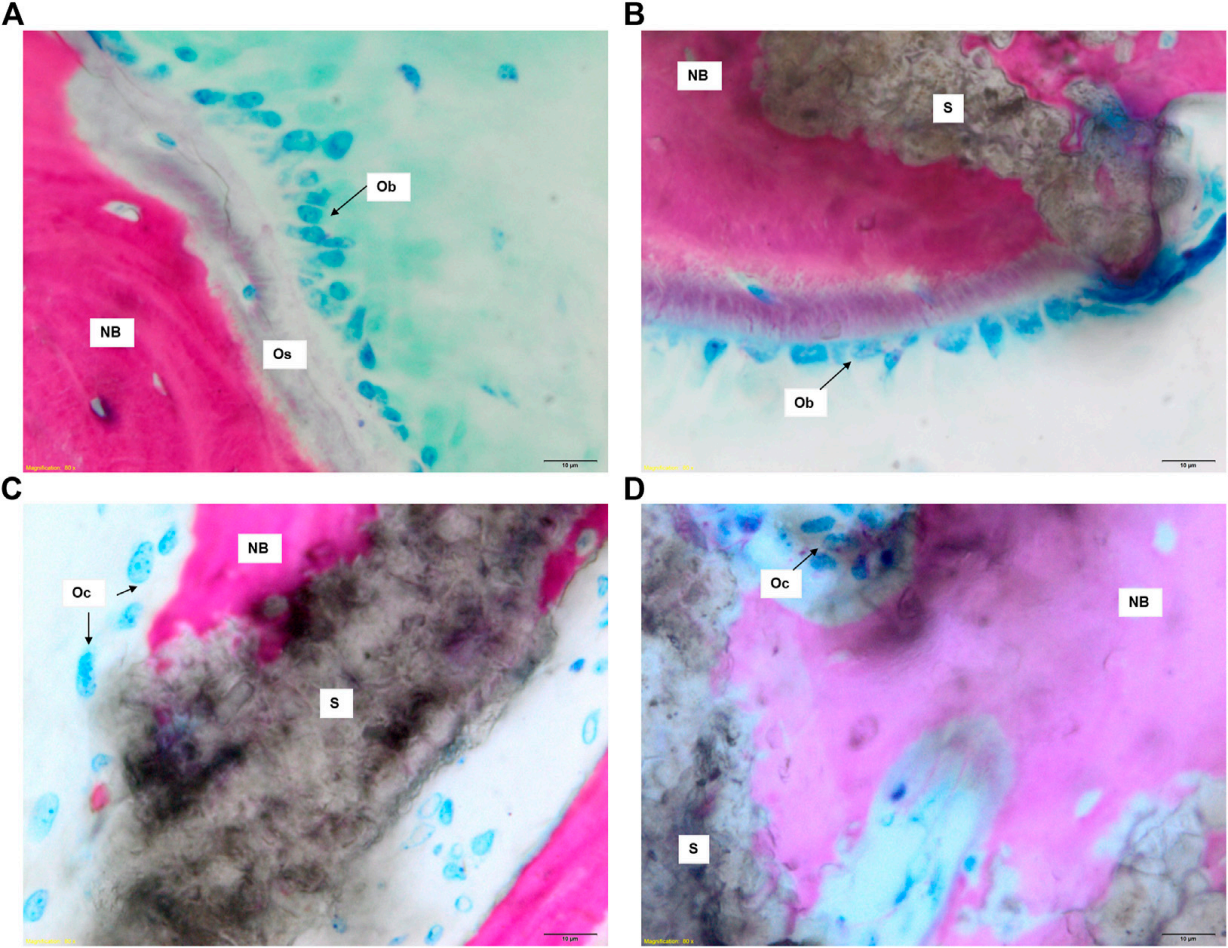
Histological images of GB-1 and GB-2 groups 6 months after reconstruction of a critical cortical defect in sheep. **(A)**, **(C)** GB-1 Group and **(B)**, **(D)** GB-2 Group. Stevenel’s blue/picro-fuchsin stain. Magnification ×80. NB: new bone; S: scaffold; Os: osteoid; Ob: osteoblast; Oc: osteoclast.

**FIGURE 10:**
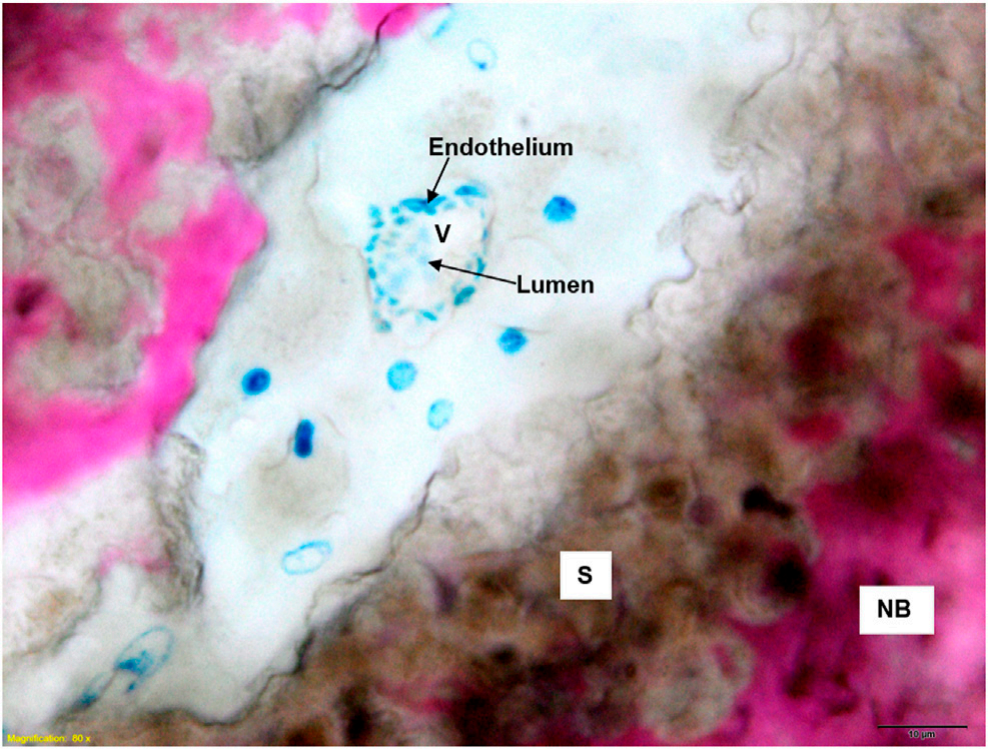
Histological images of GB-1 group 6 months after reconstruction of a critical cortical defect in sheep. NB: new bone; S: scaffold; V: vessels. Stevenel’s blue/picro-fuchsin stain. Magnification ×80.

Both GB-1 and GB-2 scaffolds were well-integrated with the host bone and a significantly higher presence of newly formed bone, with new osteonic systems, and newly formed blood vessels were seen (Figure 7) in comparison to AG group, where empty osteocytes lacunae, lack of newly formed bone tissue, and/or osteoid tissue were observed (Figure 8). GB-1 and GB-2 also showed a higher cellular activity with numerous osteoblasts and a large amount of osteoid tissue along the newly formed trabeculae (Figures 9A,B). Additionally, numerous outbreaks of osteoclastic resorption were seen on both GB-1 and GB-2 scaffolds (Figures 9C,D). Significantly higher presence of newly formed blood vessels with a well-identified lumen (containing some red blood cells) and a layer of endothelial cells were seen in GB-1 (Figure 10).

**FIGURE 11:**
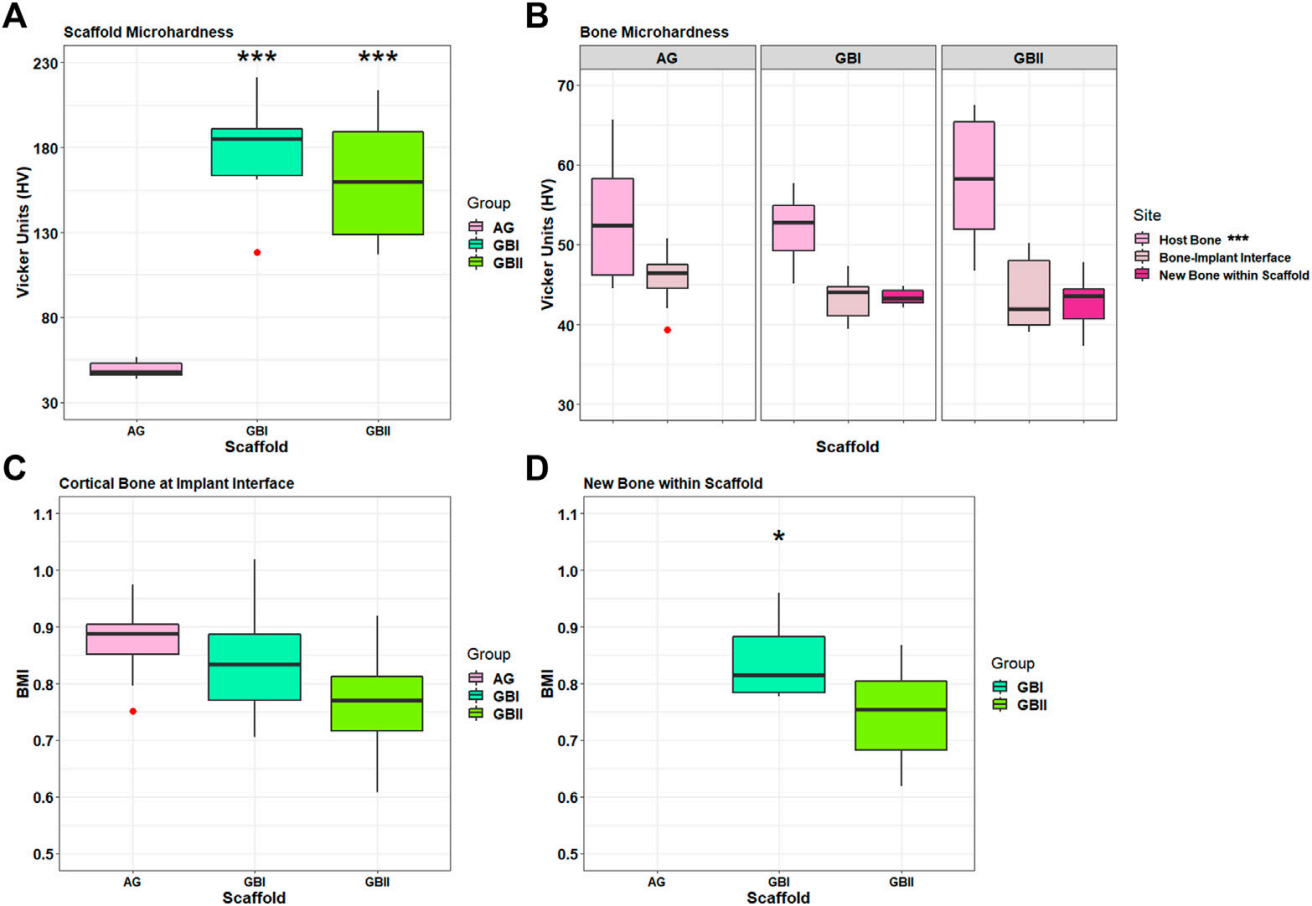
Box-plots of histomorphometric parameters OS/BS **(A)** OS.Th **(B)** and Ob. S/BS **(C)** measured on selected ROI at 6 months after surgery. Welch’s ANOVA test showed significant differences between “scaffold” groups for each histomorphometric parameter (A): OS/BS (%): F = 7.91, *p* = 0.0093 (B): OS.Th (µm): F = 10.31, *p* = 0.0038 (C): Ob. S/BS (%): F = 15.46, *p* = 0.0007. Tukey HSD test adjusted for multiple pairwise comparisons (*, *p* < 0.05, **, *p* < 0.005, ***, *p* < 0.0005).

**FIGURE 12:**
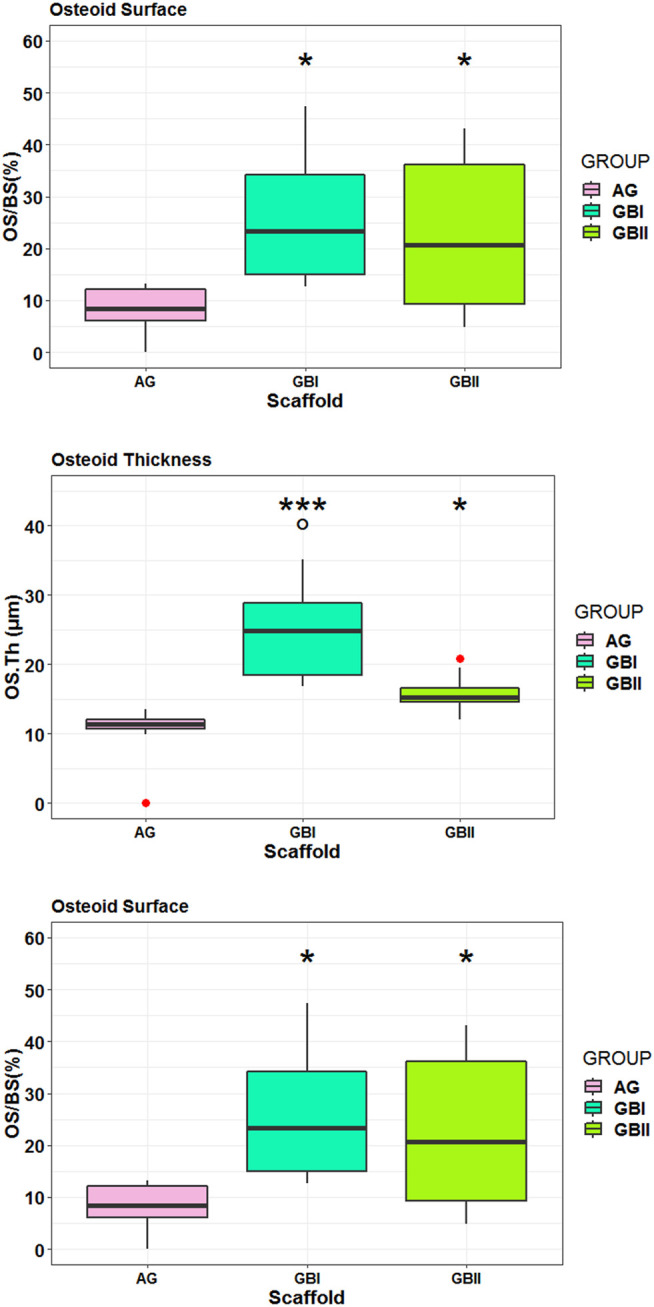
Box plots of bone microhardness. Results of GB-1, GB-2, and AG groups at 6 months after surgery. **(A)** Results of implanted scaffold: ***, *p <* 0.0005: GB-1 and GB-2 *versus* Allograft. **(B)** Results of host bone, a cortical bone at the interface with the scaffolds/AG (boneimplant interface), and newly formed bone within scaffolds measured in GB-1 and GB-2 groups. ***, *p <* 0.0005: Host bone versus boneimplant interface and new bone within scaffold. Outliers (red circle). **(C)** Bone Mineralization Index results of cortical bone at the interface with scaffold/AG and new bone within scaffolds in GB-1 and GB-2 groups. *, *p <* 0.05: GB-1 vs GB-2).

Histological data were also confirmed by histo-morphometric analyses that assessed osteoid surface (OS/BS%), osteoid thickness (OS.Th, mm) osteoblast surface (Ob.S/BS, %), and vessels/microvessels numbers. Histomorphometry showed that both GB-1 (*p* = 0.0092) and GB-2 (*p* = 0.0140) scaffolds presented higher OS/BS values in comparison to AG (Figure 11). The highest values in OS.Th and Ob. S/BS parameters were found in GB-1 scaffold and resulted significantly different from those achieved in GB-2 (OS.Th: *p* = 0.0179; Ob. S/BS: *p* = 0.0174) and AG (OS.Th: *p* = 0.0004; Ob. S/BS: *p* < 0.0005) scaffolds (Figure 12). Additionally, the OS.Th parameter was significantly higher in GB-2 than AG (*p* = 0.0064). Finally, GB-1 (15.9 ± 8.1) and GB-2 (7.9 ± 2.2) also showed the higher number of vessels/microvessels in comparison to AG, where no vessels/microvessels were seen (*p* = 0.0004 and *p* = 0.0005, respectively).

#### Microhardness

Microhardness results of non-implanted GB-1 and GB-2 scaffolds did not show any significant differences. With regard to the implanted scaffolds, significant differences were seen in microhardness in favor of GB-1 and GB-2 scaffolds compared to AG (Figure 12A). Significantly higher microhardness values of host bone in comparison with newly formed bone within scaffold and cortical bone at the interface with scaffold/AG were also seen (Figure 12B). Finally, BMI data of newly formed bone within the scaffold showed a significant lower BMI value for GB-2 in comparison with GB-1 (Figure 12C).

## Discussion

With an estimated 2-3 million grafting procedures performed each year worldwide, representing up to 10% of all skeletal reconstructive surgery (Paderni et al., 2009), the repair of long bone defects is a paramount clinical need, particularly when addressing load-bearing sites. Despite current research efforts focus on 3D bone scaffolds, none of them so far achieved satisfactory results in terms of bone regeneration and vascularization in critical segmental defects (Bose et al., 2012; Ishack et al., 2017).

The results of the present study demonstrate that, when implanted in a critical cortical defect in sheep, both GB-1 and GB-2 scaffolds were able to promote effective vascularization, osteogenesis, and osteointegration involving the whole scaffold volume, thus leading to regeneration of segmental bone defects. The scaffolds were tested without any added growth factor or osteo-inductive media, thus suggesting that their regenerative ability can be ascribed to their inherent physicochemical and structural properties, namely the bone-mimicking composition and the peculiar osteon-like porous architecture. The osteo-inductive ability of calcium phosphates and HA was the subject of intense debate so far; however, a comparative study between HA/TCP implants with different pores structures by Zhang et al. (2005) revealed that, besides composition, the coexistence of macropores and micropores in the scaffold structure is a relevant factor to improve the osteo-inductive ability. The biomorphic scaffolds tested in the present study showed not only a multi-scale porous architecture but also a complex biomimetic structural hierarchy, well resembling the osteon architecture typical of long segmental bones and exposing a lamellar nanostructure providing high specific surface for cell adhesion. Such features were previously found to promote cross-talk between osteoblasts and endothelial cells *in vitro* (Bose et al., 2012; Ishack et al., 2017); in the present large animal study, this ability resulted in extensive vascularization of the scaffold during new bone formation, thus favoring the regeneration of the whole bony defect. Furthermore, the bone-mimicking hierarchical structure of the biomorphic scaffolds can be considered as the source of its damage-tolerant mechanical behavior, similar to that of bone, and very unusual for pure ceramic materials which are usually considered as brittle (Bigoni et al., 2019; Sprio et al., 2019). Another relevant aspect in view of any potential clinical application is the scaffold’s damage-tolerant mechanical behavior that could facilitate the surgical procedure by allowing the direct screwing and application of metallic fixation plaques, thus resulting in an easy and reproducible implantation process, usually impossible when handling pure ceramic devices. Furthermore, such a mechanical ability could be a key aspect to favor the activation of mechano-transduction signaling in support to the bone remodeling process acting in a load-bearing site. In this regard, the osteopenia observed at the bone-implant interface in the animal treated with the allograft might have been determined by the lack of effective biomechanical ability.

Comparing the performance of the biomorphic GB-1 and GB-2 scaffolds, in our study, small but significant differences were seen between them. In the case of GB-1, the osteogenesis process was completed after only 3 months, achieving a complete replacement of the scaffold with new mature bone, which is a critical aspect to recover adequate biomechanical functionality. Enhanced bone regeneration with GB-1 scaffold was supported by a higher vascularization, faster formation of bone callus, and slightly improved resorption behavior, thus leading to more effective bone regeneration. This superior behavior could be ascribed to the biphasic composition of GB-1, and particularly to the presence of β-TCP phase, whose formation was induced by the doping with Mg^2+^ and Sr^2+^ ions: being more soluble than HA (Rey et al., 2014), β-TCP phase might have therefore enhanced the scaffold bio-resorbability. In addition, the presence of little amounts of Mg^2+^ and Sr^2+^ ions in GB-1 could improve new bone formation as well as favoring the regulation of the osteoblast-osteoclast activity, as previously reported, thus promoting physiological bio-resorption (Li et al., 2016; Montesi et al., 2017; Tampieri et al., 2018). A relevant aspect is that, with the except of Ca^2+^, Mg^2+^ is the most abundant divalent ion in bone, usually present in an amount of 5% during osteogenesis (particularly in the newly formed bone and young bone), while it tends to disappear in mature and ageing bone (de Bruijn et al., 1992). Thus, the presence of Mg^2+^ ions in the GB-1 scaffold could mimic the conditions found during natural osteogenesis and contribute to promote complete regeneration of the segmental bone defect. On the other hand, Sr^2+^ ions are present in traces in human bone, and following biochemical and cellular pathways similar to calcium, they are known to promote new bone formation, and furthermore, their presence was correlated to enhanced stability and mechanical strength of the newly formed bone (Habibovic and Barralet, 2011). In this respect, a synergistic effect exerted by GB-1 scaffold on bone regeneration, related to multiple ion doping and the presence of small amounts of β-TCP phase, can be hypothesized.

The experimental evidence herein provided supports the conclusion that the use of biomorphic ceramic scaffolds featuring bone-mimicking composition, morphology, and mechanics is effective to promote physiological cell behavior, triggering appropriate phenotypic differentiation, and favoring new bone formation and maturation, supported by effective vascularization. Furthermore, these findings were achieved in a clinically relevant large animal model, where biomorphic CaP scaffolds might promote the complete regeneration of load-bearing segmental bone defects created in sheep metatarsus. These results are thus very encouraging in the view of real clinical applications.

In this study, we observed that biomorphic scaffolds can overcome the limitations typical of many ceramic materials, making their use in critical orthopedic scenarios possible. In this respect, our results confirm that hydroxyapatite and tricalcium phosphate are effective materials for bone regeneration. However, very importantly, when obtained as 3D bone scaffolds, their effectiveness strongly depends on 1) their physicochemical state, i.e., particularly when they are obtained as nanostructured phases facilitating the surface interaction with cells and their bio-resorption; 2) when the scaffold shows a 3D hierarchic structure that, closely mimicking the bone architecture, can act as an instructor for cells favoring bone tissue remodeling and 3D organization; 3) when the scaffold has damage-tolerant mechanical properties, able to manage the mechanical forces *in vivo*, similarly as the natural bone tissue does. All these features could be achieved at the same time only thanks to the application of a sinter-free process, carried at ∼200°C, that allowed to retain the nanostructure and maintain the complex architectural details of the original wood template, which is a relevant issue for obtaining ideal mechanical properties and sufficient vascularization of the scaffolds.

## Conclusion

Calcium phosphate scaffolds with hierarchically organized structure, developed through a biomorphic transformation process preventing the sintering treatment, showed good results in terms of safety, osteo-inductivity, osteo-conductivity, osteo-integration, vascularization, and mechanical performance, when tested in a load-bearing segmental bone defect in a large animal model. These findings suggest that biomorphic scaffolds presenting high mimicry with bone under a physico-chemical, structural, and mechanical perspective are promising candidates for future clinical applications addressing regeneration of load-bearing critical bone defects, which is a still unmet clinical need, also due to critical limitations of devices currently available for this specific application.

## Data Availability

The raw data supporting the conclusion of this article will be made available by the authors, without undue reservation.

## References

[B1] Bigoni D. Cavuoto R. Misseroni D. Paggi M. Ruffini A. Sprio S. (2019). Editorial Board. Mater. Today Adv. 3 (5), 100032. 10.1016/s2590-0498(19)30106-7 PMC7083766 32211602

[B2] Bose S. Roy M. Bandyopadhyay A. (2012). Recent Advances in Bone Tissue Engineering Scaffolds. Trends Biotechnol. 30 (10), 546–554. 10.1016/j.tibtech.2012.07.005 22939815 PMC3448860

[B3] de Bruijn J. D. Klein C. P. A. T. de Groot K. van Blitterswijk C. A. (1992). The Ultrastructure of the Bone-Hydroxyapatite Interfacein Vitro. J. Biomed. Mater. Res. 26 (10), 1365–1382. 10.1002/jbm.820261008 1331114

[B4] Ebrahimi A. Nejadsarvari N. Ebrahimi A. Rasouli H. R. (2017). Early Reconstructions of Complex Lower Extremity Battlefield Soft Tissue Wounds. World J. Plast. Surg. 6 (3), 332–342. 29218283 PMC5714979

[B5] Filardo G. Roffi A. Fey T. Fini M. Giavaresi G. Marcacci M. (2020). Vegetable Hierarchical Structures as Template for Bone Regeneration: New Bio‐ceramization Process for the Development of a Bone Scaffold Applied to an Experimental Sheep Model. J. Biomed. Mater. Res. 108 (3), 600–611. 10.1002/jbm.b.34414 31095882

[B6] Giannoudis P. V. Atkins R. (2007). Management of Long-Bone Non-unions. Injury 38 (Suppl. 2), S1–S2. 10.1016/s0020-1383(07)80002-7 17920411

[B7] Gibson L. J. Ashby M. F. (1999). Cellular Ceramics – Structure and Properties. Editors Clarke DR Suresh S Ward IM. 2nd ed (UK: Cambridge University Press).

[B8] Ginebra M. P. Espanol M. Maazouz Y. Bergez V. Pastorino D. (2018). Bioceramics and Bone Healing. EFORT Open Rev. 3 (5), 173–183. 10.1302/2058-5241.3.170056 29951254 PMC5994622

[B9] Habibovic P. Barralet J. E. (2011). Bioinorganics and Biomaterials: Bone Repair. Acta Biomater. 7 (8), 3013–3026. 10.1016/j.actbio.2011.03.027 21453799

[B10] Habraken W. Habibovic P. Epple M. Bohner M. (2016). Calcium Phosphates in Biomedical Applications: Materials for the Future? Mater. Today 19 (2), 69–87. 10.1016/j.mattod.2015.10.008

[B11] Hannink G. Arts J. J. C. (2011). Bioresorbability, Porosity and Mechanical Strength of Bone Substitutes: what Is Optimal for Bone Regeneration? Injury 42 (Suppl. 2), S22–S25. 10.1016/j.injury.2011.06.008 21714966

[B12] Huang C. Ogawa R. (2002). Mechanotransduction in Bone Repair and Regeneration. FASEB J. 24 (10), 3625–3632. 10.1096/fj.10-157370 20505115

[B13] Hutmacher D. W. Sittinger M. Risbud M. V. (2004). Scaffold-based Tissue Engineering: Rationale for Computer-Aided Design and Solid Free-form Fabrication Systems. Trends Biotechnol. 22 (7), 354–362. 10.1016/j.tibtech.2004.05.005 15245908

[B14] Ishack S. Mediero A. Wilder T. Ricci J. L. Cronstein B. N. (2017). Bone Regeneration in Critical Bone Defects Using Three-Dimensionally Printed β-tricalcium Phosphate/hydroxyapatite Scaffolds Is Enhanced by Coating Scaffolds with Either Dipyridamole or BMP-2. J. Biomed. Mater. Res. 105 (2), 366–375. 10.1002/jbm.b.33561 PMC5344038 26513656

[B15] LeGeros R. Z. (2002). Properties of Osteoconductive Biomaterials: Calcium Phosphates. Clin. Orthopaedics Relat. Res. 395, 81–98. 10.1097/00003086-200202000-00009 11937868

[B16] Li M. He P. Wu Y. Zhang Y. Xia H. Zheng Y. (2016). Stimulatory Effects of the Degradation Products from Mg-Ca-Sr alloy on the Osteogenesis through Regulating ERK Signaling Pathway. Sci. Rep. 6 (6), 32323. 10.1038/srep32323 27580744 PMC5007487

[B17] Liu H. Slamovich E. B. Webster T. J. (2006). Less Harmful Acidic Degradation of Poly(lactic-Co-Glycolic Acid) Bone Tissue Engineering Scaffolds through Titania Nanoparticle Addition. Int. J. Nanomedicine 1 (4), 541–545. 10.2147/nano.2006.1.4.541 17722285 PMC2676635

[B18] Montesi M. Panseri S. Dapporto M. Tampieri A. Sprio S. (2017). Sr-substituted Bone Cements Direct Mesenchymal Stem Cells, Osteoblasts and Osteoclasts Fate. PLoS One 12 (2), e0172100. 10.1371/journal.pone.0172100 28196118 PMC5308610

[B19] Paderni S. Terzi S. Amendola L. (2009). Major Bone Defect Treatment with an Osteoconductive Bone Substitute. Musculoskelet. Surg. 93 (2), 89–96. 10.1007/s12306-009-0028-0 19711008

[B20] R Team (2002). A Language and Environment for Statistical Computing. Computing Vienna, Austria, 1(2). CO. 083[3097:CFHIWS]2.0. 10.1890/0012-9658

[B21] Rey C. Combes C. Drouet C. Cazalbou S. Grossin D. Brouillet F. (2014). Surface Properties of Biomimetic Nanocrystalline Apatites; Applications in Biomaterials. Prog. Cryst. Growth Characterization Mater. 60 (3-4), 63–73. 10.1016/j.pcrysgrow.2014.09.005

[B22] Roffi A. Krishnakumar G. S. Gostynska N. Kon E. Candrian C. Filardo G. (2017). The Role of Three-Dimensional Scaffolds in Treating Long Bone Defects: Evidence from Preclinical and Clinical Literature-A Systematic Review. Biomed. Res. Int. 2017, 8074178. 10.1155/2017/8074178 28852649 PMC5567443

[B23] Sprio S. Panseri S. Montesi M. Dapporto M. Ruffini A. Dozio S. M. (2019). Hierarchical Porosity Inherited by Natural Sources Affects the Mechanical and Biological Behaviour of Bone Scaffolds. J. Eur. Ceram. Soc. 40 (4), 1717–1727.

[B24] Stegen S. van Gastel N. Carmeliet G. (2015). Bringing New Life to Damaged Bone: the Importance of Angiogenesis in Bone Repair and Regeneration. Bone 70, 19–27. 10.1016/j.bone.2014.09.017 25263520

[B25] Tampieri A. Ruffini A. Ballardini A. Montesi M. Panseri S. Salamanna F. (2018). Heterogeneous Chemistry in the 3-D State: an Original Approach to Generate Bioactive, Mechanically-Competent Bone Scaffolds. Biomater. Sci. 7 (1), 307–321. 10.1039/c8bm01145a 30468436

[B26] Tampieri A. Sprio S. Ruffini A. Celotti G. Lesci I. G. Roveri N. (2009). From wood to Bone: Multi-step Process to Convert wood Hierarchical Structures into Biomimetic Hydroxyapatite Scaffolds for Bone Tissue Engineering. J. Mater. Chem. 19, 4973–4980. 10.1039/b900333a

[B27] Tomlinson P. B. Fisher J. B. Spangler R. E. Richer R. A. (2001). Stem Vascular Architecture in the rattan palm Calamus (Arecaceae-Calamoideae-Calaminae). Am. J. Bot. 88 (5), 797–809. 10.2307/2657032 11353705

[B28] Zhang Z. Kurita H. Kobayashi H. Kurashina K. (2005). Osteoinduction with HA/TCP Ceramics of Different Composition and Porous Structure in Rabbits. Oral Sci. Int. 2 (2), 85–95. 10.1016/s1348-8643(05)80011-4

